# Postural assessment of children with congenital Zika syndrome and caregivers in the home environment: a cross-sectional pilot study

**DOI:** 10.1590/1516-3180.2023.0354.R2.29042025

**Published:** 2025-10-13

**Authors:** Janiele de Sales Tavares, Thamyris de Sales Regis, Gabriela Lopes Gama, José Geraldo Ribeiro Gregório, Jousilene de Sales Tavares, Adriana Melo, Daniel Scherer

**Affiliations:** I Physiotherapist, Researcher, Instituto Assistencial Professor Joaquim Amorim Neto, Campina Grande (PB), Brazil.; II Physiotherapist, Researcher, Instituto Assistencial Professor Joaquim Amorim Neto, Campina Grande (PB), Brazil.; III Professor, Physical Therapy Department, Universidade Federal de Juiz de Fora (UFJF), Governador Valadares (MG), Brazil.; IV Speech Therapist, Researcher, Instituto Assistencial Professor Joaquim Amorim Neto, Campina Grande (PB), Brazil.; V Physical Education Professional, Researcher, Instituto Assistencial Professor Joaquim Amorim Neto, Campina Grande (PB), Brazil.; VI Physician, Researcher, Instituto Assistencial Professor Joaquim Amorim Neto, Campina Grande (PB), Brazil.; VII Bachelor’s in Computer Science, Professor, Center for Strategic Technologies in Health (NUTES), Universidade Estadual da Paraíba (UEPB), Campina Grande (PB), Brazil.

**Keywords:** Posture., Zika virus infection., Child care., Respite care., Microcephaly., Caring mothers., Daily care., Everyday tasks.

## Abstract

**BACKGROUND:**

Children with congenital Zika syndrome (CZS) present severe motor impairment that hinders their caregivers’ positioning during activities of daily living (ADLs).

**OBJECTIVE:**

To assess the posture of children with CZS and their caregivers during ADLs in the home environment.

**DESIGN AND SETTING:**

A cross-sectional pilot study conducted in Campina Grande (PB), Brazil.

**METHODS:**

Nine children with CZS (mean age = 36.77 ± 2.94 months) and their caregivers (n = 9, mean age = 27 years) were assessed. Data were collected at the support home of a center for children with microcephaly in Northeast of Brazil. For postural assessment, children and their caregivers were filmed while performing ADLs in the living room and kitchen of the support home.

**RESULTS:**

During the environmental interaction, all children predominantly maintained a sitting position, exhibiting neck and trunk asymmetry; 77.8% (n = 7) showed inadequate postures with elevated arms and shoulders, and none maintained the ankles in a neutral position with supported feet. During feeding, 88.9% (n = 8) of children were positioned on the lap of caregivers, 88.9% (n = 8) exhibited neck and trunk asymmetry, 66.7% (n = 6) displayed inadequate upper-limb posture, and none maintained their ankles in a neutral position. During this activity, 66.7% (n = 6) of caregivers presented with trunk and neck asymmetry, 66.7% (n = 6) did not provide support for the upper limbs, and 55.6% (n = 5) did not maintain their knees flexed at 90^o^.

**CONCLUSIONS:**

Children with CZS and their caregivers present with inadequate postures during ADLs in the home environment, which may represent health risks.

## INTRODUCTION

 Congenital Zika syndrome (CZS), which was initially described in 2016, is characterized by neurodevelopmental impairments due to intrauterine Zika virus infections.^1^ Microcephaly is the primary and most evident clinical manifestation of CZS; nevertheless, children with this pathology may present with other neurological abnormalities such as juxtacortical calcifications, cortical malformations, ventriculomegaly, and cerebellar and brainstem hypoplasia.^2-5^

 Upon physical examination, children with CZS often show craniofacial disproportions, occipital bone protrusion, closed fontanelles at birth, and excessive or folded scalp skin.^5,6^ Other impairments become apparent with age, including severe intellectual and motor disabilities, altered muscle tone, irritability, intractable epilepsy, dysphagia, and anomalies in the visual and auditory systems.^5,7-9^

 Neurological impairment in children with CZS may delay or impair motor development. Wheeler et al. (2018) assessed 47 children with CZS aged 16 months and reported that one child could maintain an upright position without support, whereas approximately 25% could roll or maintain a quadruped position without help.^10^ Therefore, children with CZS often remain seated, lying down, or held by caregivers for long periods. By contrast, caregivers of children with CZS frequently experience musculoskeletal pain, which can compromise their quality of life.^2,11,12^

 Analyzing the posture of children with CZS and their caregivers during activities of daily living (ADLs) is crucial for understanding the risk of osteoarticular deformities in children and musculoskeletal pain in caregivers, aiding in the selection of adequate therapeutic interventions. To date, the postures of children with CZS and their caregivers during ADLs have not been analyzed in detail. Performing this analysis in everyday environments can reveal difficulties experienced by children and caregivers. 

## OBJECTIVE

This study aimed to assess the posture of children with CZS and their caregivers during ADLs in the home environment.

## METHODS

 This cross-sectional pilot study followed the Strengthening the Reporting of Observational Studies in Epidemiology (STROBE)^13^ guidelines. The study was developed by the Instituto Assistencial Professor Joaquim Amorim Neto (IPESQ) and the Center for Strategic Technologies in Health at the Universidade Estadual da Paraíba (UEPB). Data were collected in February and March 2020. Informed consent was obtained from all children’s parents or legal guardians. All procedures were conducted in accordance with the Declaration of Helsinki, and the study was approved by the Institutional Review Board of Hospital Universitário Alcides Carneiro on February 21, 2020 (protocol number 26305819.2.0000.5182). 

### Sample

 Children and their caregivers were recruited from a support center for children with microcephaly linked to the IPESQ in Campina Grande (PB), Brazil, using a non-probabilistic technique. Since 2015, this center has provided multidisciplinary care for children with CZS. The IPESQ health team comprises trained and experienced medical doctors, nurses, physical therapists, speech therapists, and occupational therapists. 

 Children assisted in this center are from several Brazilian states and other countries in Latin America and Africa. Thus, mothers reside in a support home while their children undergo rehabilitation interventions. When study was conducted a total of 71 children with CZS were assisted by the IPESQ, 24 of whom resided in the support home. All children evaluated in the present study resided in the support home when this study was conducted. 

 Samples were obtained using non-probability convenience sampling. Children were included in this study if they (1) had CZS diagnosed by reverse transcription polymerase chain reaction (RT-PCR) or imaging findings in the first months of life (computed tomography or magnetic resonance imaging), (2) were at least one year of age, and (3) required a caregiver to perform ADLs. Children who were not present for data collection and had osteoarticular deformities that could limit the sitting position were excluded. 

### Data collection and assessment procedures

 First, general data on children (age, height, sex, and weight) and caregivers (age, sex, educational level, income, habitation, symptoms of Zika virus infection in pregnancy, trimester with Zika virus infection symptoms in pregnancy, type of delivery, and experience of caring for children with neurological impairment) were collected using IPESQ records and interviews with the caregivers. Motor impairment, ability to control the neck and trunk, and muscle tone were also assessed. 

 The Gross Motor Function Classification System (GMFCS), which classifies the level of motor impairment from level I (minimum impairment) to level V (severe impairment)^14-16^, was employed to assess locomotion capacity and functionality according to age. 

 The Gross Motor Function Measure (GMFM) was used to assess the ability to control the neck and trunk. The GMFM assesses gross motor function during 88 tasks, with the score ranging from 0 (unable to initiate the task) to 3 (able to complete the task) for each task.^17^ However, in this study, only item 22 (sitting on the carpet, supported on the chest by the therapist: raises the head in the midline, holds for 10 seconds) and item 23 (sitting on the carpet with the arms supported: maintains for 5 seconds) were used.^18^

 The axial and proximal appendicular muscle tone of the children was assessed using the pull-to-sit maneuver, ventral suspension, scarf sign, and shoulder suspension.^19^ For each maneuver, the children’s responses were classified as positive (+, hypotonia) or negative (-, no hypotonia) according to the expected. Children who presented with a positive classification for at least one maneuver were considered to have axial hypotonia. 

 Due to resistance to passive movement, the proximal appendicular muscle tone was assessed using the Modified Ashworth Scale (MAS). The MAS classifies muscular resistance to passive movement into six levels: 0 (no increase in muscle tone) to 4 (rigid affected segment without movement).^20^

 Postural assessment of caregivers and children during ADLs was performed after the physical examination. The assessment was conducted in the living room and kitchen of the support center for mothers, where caregivers and children performed most ADLs. In the living room, the postures of children were assessed while sitting during activities such as watching television, manipulating objects, and tasks stimulating the motor system and requiring interaction with the environment. In the kitchen, the postures of children and caregivers were assessed during feeding while considering task execution, positioning, and movement removal. 

 Films were recorded using a Canon EOS Rebel SL2 video camera positioned in front of the caregivers and children prior to the tasks. Thus, the researcher did not interact with the caregivers or children during filming to capture the postures as closely as possible to reflect daily reality. 

 The films were then analyzed according to a questionnaire adapted from the Brazilian version of the International Standard Organization (NBR ISO) 11,226, entitled "Ergonomics — Evaluation of static work postures – Rapid evaluation".^21^ NBR ISO 11,226 establishes ergonomic recommendations for different work tasks, justifying the adaptation. Thus, we used the second stage of NBR ISO 11,226 by adapting 17 questions into 12–21 dichotomous questions (yes or no) for each body segment (neck, trunk, upper limbs, and lower limbs). The questions encompassed the essential elements for adequate posture in each task. 

 Regarding upper- and lower-limb assessments, the worst-positioned limb was considered the most overloaded limb. This analysis allowed for the identification of possible postural deviations, inadequacies in the use of equipment (e.g., positioning chairs), and health risks of postures. The absence of risk was considered when all questions in the questionnaire were answered with "yes." If any question presented the answer "no," the task was considered a health risk. 

### Statistical analysis

 Initially, a specific database was created using REDCap software. Descriptive analysis was performed using measures of central tendency (mean) and dispersion (standard deviation) for continuous variables, such as the age, weight, and height of children and caregivers. In addition, absolute and relative frequencies of categorical variables (e.g., health risks from posture and microcephaly at birth) were determined. 

## RESULTS

 Nine children aged 34–44 months (36.77 ± 2.94 months) were assessed, with five (55.5%) female children. Eight (88.8%) children were classified as GMFCS level V, and five (55.5%) had axial hypotonia and proximal appendicular hypertonia. All caregivers were mothers aged 23–35 years (27 ± 4.69 years), with three (33.3%) reporting a complete higher education (**Table 1**). 

**Table 1:** Characterization of children and caregivers

**Variables**		**n (%)**	**Mean ± SD**	**Range**
**Characteristics of children**				
Age (months)			36.26 ± 3.79	34–44
Sex				
	Female	4 (44.4)		
	Male	5 (55.5)		
Weight (kg)			11.54 ± 1.56	9–12.63
Height (cm)			90.27 ± 3.86	86–97
Microcephaly				
	Yes	8 (88.8)		
	No	1(11.1)		
*GMFCS level*				
	IV	1 (11.1)		
	V	8 (88.8)		
*Item 22 GMFM*				
	1 – Initiate the task	1 (11.1)		
	2 – Partially completes the task	2 (22.2)		
	3 – Completes the task	6 (66.6)		
*Item 23 GMFM*				
	0 – Unable to initiate the task	4 (44.4)		
	3 – Completes the task	5 (55.5)		
*Muscle tone classification*				
	No axial hypotonia and no proximal appendicular hypertonia	1 (11.1)		
	No axial hypotonia and proximal appendicular hypertonia	3 (33.3)		
	Axial hypotonia and proximal appendicular hypertonia	5 (55.5)		
**Characteristics of caregivers**				
Age (years)			27 ± 4.690	22–35
Weight (kg)			67.44 ± 11.640	53–86
Height (m)			1.56 ± 0.057	1.50–1.64
Educational level				
	Complete high school	6 (66.6)		
	Complete higher education	3 (33.3)		
Experience of caring (years)			7 ± 4.18	4–16
Income (*reais* )			646.91 ± 493.69	50–1.625
Habitation				
	Urban area	8 (88.8)		
	Countryside	1 (11.1)		
Symptoms of Zika virus infection in pregnancy				
	Yes	8 (88.8)		
	No	1 (11.1)		
Trimester with Zika virus infection symptoms in pregnancy				
	First trimester	6 (66.6)		
	Second trimester	2 (22.2)		
	No symptoms	1 (11.1)		
Type of delivery				
	Vaginal delivery	5 (55.5)		
	Cesarean section	4 (44.4)		

GMFCS = Gross Motor Function Classification System; GMFM = Gross Motor Function Measure; CZS = congenital Zika syndrome.

### Postural assessment of children

 During the environmental interaction activities, all children predominantly maintained a sitting position, exhibiting neck and trunk asymmetry. Seven children (77.8%) demonstrated anterior neck and trunk inclination. Regarding upper-limb posture, none maintained their elbows at a 90^o^ angle, and seven exhibited inadequate postures with elevated arms and shoulders. The wrist showed a less inadequate posture, with only one child (11.1%) displaying a radial deviation. Lower-limb analysis revealed that none of the children maintained their ankles in a neutral position with supported feet, six children (66.7%) exhibited hip abduction, and five children (55.6%) showed internal hip rotation. **Table 2** presents the details of these findings. 

**Table 2:** Postural assessment of children during environmental interactions

**Evaluation criteria**	**Yes n (%)**	**No n (%)**
**Assessment of neck and trunk**			
Posture of the trunk and neck are symmetrical		9 (100)
Trunk flexion above 20^o^?	-	9 (100)
No trunk inclination?	2 (22.2)	7 (77.8)
No neck tilt?	2 (22.2)	7 (77.8)
Trunk remains supported?	2 (22.2)	7 (77.8)
**Assessment of the most burdened upper limb**			
No inappropriate arm postures?	2 (22.2)	7 (77.8)
No shoulder lift?	2 (22.2)	7 (77.8)
Arms raised below 20^o^ without support?	4 (44.4)	5 (55.6)
Arms with elevation above 60^o^ and with full support?	-	9 (100)
No extreme flexion and extension or rotation of the elbow?	2 (22.2)	7 (77.8)
Elbow flexed at 90^o^?	-	9 (100)
No extreme wrist deviation?	8 (88.9)	1 (11.1)
**Assessment of the most burdened lower limb**			
No kneeling and crouching posture?	9 (100)	-
No hip external rotation?	8 (88.9)	1 (11.1)
No hip internal rotation?	4 (44.4)	5 (55.6)
Hip remains at 90^o^?	6 (66.7)	3 (33.3)
Knee remains at 90^o^?	5 (55.6)	4 (44.4)
No knee abduction?	3 (33.3)	6 (66.7)
No knee abduction?	8 (88.9)	1 (11.1)
Ankle remains in neutral position and feet supported?	-	9 (100)

**Table 3** describes the criteria and results of postural assessments of the children during feeding. During feeding, eight children (88.9%) were positioned on the caregivers’ lap, one child (11.1%) was in a wheelchair, and none were in a feeding chair. Analysis of images depicting the execution of this task revealed that eight children (88.9%) exhibited neck and trunk asymmetry, whereas seven children (77.8%) presented neck and trunk inclination. Six children (66.7%) displayed inadequate upper-limb posture, and all children had their elbows flexed at a 90^o^ angle. As for lower limbs, none of the children maintained their ankles in the neutral position with their feet supported during feeding. 

**Table 3:** Postural assessment of children during feeding

**Evaluation criteria**	**Yes n (%)**	**No n (%)**
**Assessment of neck and trunk**			
Posture of the trunk and neck are symmetrical	1 (11.1)	8 (88.9)
Trunk flexion above 20^o^?	-	9 (100)
No trunk inclination?	2 (22.2)	7 (77.8)
No neck tilt?	2 (22.2)	7 (77.8)
Trunk remains supported?	2 (22.2)	7 (77.8)
**Assessment of the most burdened upper limb**			
No inappropriate arm postures?	3 (33.3)	6 (66.7)
No shoulder lift?	7 (77.8)	2 (22.2)
Arms raised below 20^o^ without support?	6 (66.7)	3 (33.3)
Arms with elevation above 60^o^ and with full support?	9 (100)	-
No extreme flexion and extension or rotation of the elbow?	6 (66.7)	3 (33.3)
Elbow flexed at 90^o^?		9 (100)
No extreme wrist deviation?	9 (100)	-
**Assessment of the most burdened lower limb**			
No kneeling and crouching posture?	9 (100)	-
No hip external rotation?	7 (77.8)	2 (22.2)
No hip internal rotation?	8 (88.9)	1 (11.1)
Hip remains at 90^o^?	5 (55.6)	4 (44.4)
Knee remains at 90^o^?	5 (55.6)	4 (44.4)
No knee abduction?	6 (66.7)	3 (33.3)
No knee abduction?	8 (88.9)	1 (11.1)
Ankle remains in neutral position and feet supported?	-	9 (100)

### Postural assessment of caregivers

 Six caregivers (66.7%) presented with trunk and neck asymmetry during feeding. All caregivers presented neck inclination, and seven (77.8%) presented trunk inclination. Caregivers also showed altered upper-limb positioning, such as arm elevation (n = 9), lack of support (n = 6), and no elbow flexion at 90^o^ (n = 9). Regarding the lower limbs, no caregivers maintained their ankles in a neutral position with supported feet, and five caregivers (55.6%) did not maintain their knees flexed at 90^o^ (**Table 4**). 

**Table 4:** Postural assessment of caregivers during feeding

**Evaluation criteria**	**Yes n (%)**	**No n (%)**
**Assessment of neck and trunk**			
Posture of the trunk and neck are symmetrical	3 (33.3)	6 (66.7)
Trunk flexion above 20^o^?	-	9 (100)
No trunk inclination?	2 (22.2)	7 (77.8)
No neck tilt?	-	9 (100)
Trunk remains supported?	5 (55.6)	4 (44.4)
**Assessment of the most burdened upper limb**			
No inappropriate arm postures?	-	9 (100)
No shoulder lift?	4 (33.3)	6 (66.7)
Arms raised below 20^o^ without support?	4 (33.3)	6 (66.7)
Arms with elevation above 60^o^ and with full support?	-	9 (100)
No extreme flexion and extension or rotation of the elbow?	6 (66.7)	3 (33.3)
Elbow flexed at 90^o^?	-	9 (100)
No extreme wrist deviation?	7 (77.8)	2 (22.2)
**Assessment of the most burdened lower limb**			
No kneeling and crouching posture?	9 (100)	-
No hip external rotation?	6 (66.7)	3 (33.3)
No hip internal rotation?	8 (88.9)	1 (11.1)
Hip remains at 90^o^?	5 (55.6)	4 (44.4)
Knee remains at 90^o^?	4 (44.4)	5 (55.6)
No knee abduction?	5 (55.6)	4(44.4)
No knee abduction?	8 (88.9)	1 (11.1)
Ankle remains in neutral position and feet supported?	-	9 (100)

 Regarding the posture adopted for positioning or removing children from the feeding position, caregivers reported trunk inclination and shoulder elevation. Eight caregivers (88.9%) did not perform hip or knee flexion and no caregivers presented with lateral wrist deviation. The data related to this analysis are presented in **Table 5**. 

**Table 5:** Postural assessment of caregivers during laying and removal of the child from feeding posture

**Evaluation criteria**	**Yes n (%)**	**No n (%)**
**Assessment of neck and trunk**			
Are trunk and neck symmetrical?	3 (33.3)	6 (66.7)
Trunk flexion below 20^o^?	3 (33.3)	6 (66.7)
No trunk hyperextension when removing the child?	3 (33.3)	6 (66.7)
No trunk inclination?	-	9 (100)
No neck tilt?	4 (44.4)	5 (55.6)
Flexion of legs and keep torso straight when removing the child?	1 (11.1)	8 (88.9)
**Assessment of the most burdened upper limb**			
No inappropriate arm postures?	-	9 (100)
No shoulder lift?	-	9 (100)
No extreme flexion and extension?	8 (88.9)	1 (11.1)
No extreme wrist deviation?	6 (100)	-
**Assessment of the most burdened lower limb**			
No kneeling and crouching posture?	6 (100)	-
Flexion of the knees and hips when removing the child?	1(11.1)	8 (88.9)

## DISCUSSION

 In the present study, monitoring children with CZS during ADLs in a home environment allowed for the identification of inadequate postures in children and caregivers. Specifically, most children exhibited neck and trunk asymmetry and foot misalignment during the tasks. During feeding, caregivers frequently presented with postural inadequacies of the trunk and neck. In addition, most caregivers demonstrated postural inadequacies in all body segments during positioning and removal of children from the feeding position. The results presented here demonstrate the postural inadequacies experienced by children with CZS and their caregivers during ADLs. These postures may represent risks to children’s and caregivers’ health; however, this hypothesis should be investigated in future studies. 

 Motor impairment often hampers the ability to sit, stand, and perform ADLs independently.^22-24^ The motor impairment of children with CZS has been extensively discussed in the literature since the description of the first cases.^8,23,24^ Melo et al. reported that 81% of children with CZS had severe motor impairments, classified as GMFCS level V.^8^ Similarly, in this study, children were classified as GMFCS levels IV and V, requiring help to perform ADLs. 

 Inadequate positioning, severe motor impairment, altered muscle tone, muscle weakness, impaired motor coordination, and excessive agonist or antagonist co-activation may cause secondary neuromotor complications (e.g., shortening and contractures) in children with neurological impairment.^25^ High GMFCS levels accelerate these complications in children with CZS, who are also at risk of multiple contractures and postural inadequacies.^ 26,27^ Likewise, in the children assessed in this study, impaired motor function and inadequate positioning during ADLs suggest an increased risk of developing secondary osteoarticular deformities, compromising motor function and quality of life of children and caregivers. 

 Casey et al. evaluated contractures in children with different GMFCS levels. In their study, children with GMFCS levels IV and V presented with contractures mostly in the knee joint, followed by those in the hip joint. This finding may be due to the long sitting time of these children.^27^ However, the present study did not observe significant knee joint asymmetry, possibly because a detailed assessment of this joint was hindered as the children remained seated during the tasks. 

 Considering the risk of secondary complications related to positioning and identified inadequacies, addressing these postures is crucial in rehabilitation programs for children with CZS. In this sense, proper seating has been suggested as a strategy for normalizing muscle tone, reducing the influence of abnormal reflexes, improving motor control and functionality, ensuring skin integrity, and preventing the development and emergence of deformities.^28^ Despite the wide range of adaptive equipment available in the market to promote positioning and functionality for children with postural control deficits, most children in this study were fed at the laps of caregivers. This observation may be linked to the socioeconomic conditions of the mothers (Sa et al.), considering the cost of equipment.^29^

 Since the Zika virus infection epidemic, most mothers of children with CZS are young and have low income.^29^ Over the years, this situation has worsened because many women need to abandon paid work to care for their children or have been abandoned by their partners. Moreover, mothers of children with CZS are often the only ones responsible for supporting and caring for their children.^29^ Caring for children with physical disabilities broadly affects caregivers’ quality of life. Previous studies have described the impact on the mental and physical health of mothers of children with CZS, especially due to the dependence of children in performing ADLs.^30,31^

 A study conducted on 63 mothers of children with CZS reported musculoskeletal pain in 93.7% of mothers, with the lower back, thoracic, and cervical spine having the highest incidence of pain. The study demonstrated that pain complaints were directly related to lower scores on the quality-of-life assessment.^11^ Moreover, inadequate postures adopted by mothers may be associated with the musculoskeletal pain reported in other studies. Furthermore, this indicates that caring for children with CZS overlaps with the mothers’ health, which is impaired by the lack of auxiliary devices and health education strategies. Thus, there is a need to implement health education strategies involving the daily positioning of mothers and children to reduce health risks. 

 This study has several limitations. First, the sample size and investigated tasks were small. However, they allowed for maximum similarity between the daily lives of the caregivers and the environment. Second, the assessment procedure (film analysis) was subjective; nonetheless, this limitation was minimized by using trained evaluators and the same evaluator for all analyses. 

 By contrast, this study has strength in that it evaluated children with CZS in a familiar environment, as human performance cannot be analyzed in isolation from environmental conditions. ADLs depend on multiple factors related to the individual, the environment, and the task.^32^

 These findings highlight the need for further research using larger sample sizes and health education strategies to correctly positioning children and caregivers during ADLs. Moreover, lowcost and accessible assistive devices are essential to ensure adequate positioning of children and caregivers, reducing health risks during ADLs. 

## CONCLUSION

 Postural analysis of children with CZS and their caregivers revealed inadequate postures related to improper positioning of children. By repetition, children may develop secondary osteoarticular deformities, compromising their motor function and quality of life and increasing health risks. Additionally, all caregivers could not maintain adequate postural alignment, potentially increasing health risks. 
